# Development and Validation of a Sensitive, Specific and Reproducible UPLC-MS/MS Method for the Quantification of OJT007, A Novel Anti-Leishmanial Agent: Application to a Pharmacokinetic Study

**DOI:** 10.3390/ijerph18094624

**Published:** 2021-04-27

**Authors:** Maria Rincon Nigro, Jing Ma, Ololade Tosin Awosemo, Huan Xie, Omonike Arike Olaleye, Dong Liang

**Affiliations:** 1 Department of Pharmaceutical Sciences, College of Pharmacy and Health Sciences, Texas Southern University, Houston, TX 77004, USA; m.rinconnigro7799@student.tsu.edu (M.R.N.); jing.ma@tsu.edu (J.M.); ololade.awosemo@bms.com (O.T.A.); huan.xie@tsu.edu (H.X.); omonike.olaleye@tsu.edu (O.A.O.); 2 Department of Clinical Pharmacology Analysis and Reporting, Bristol Myers Squibb, Princeton, NJ 08543, USA

**Keywords:** OJT007, UPLC-MS/MS, pharmacokinetics, leishmaniasis, rats

## Abstract

OJT007 is a methionine aminopeptidase 1 (MetAP1) inhibitor with potent anti-proliferative effects against *Leishmania Major*. In order to study its pharmacokinetics as a part of the drug development process, a sensitive, specific, and reproducible ultra-high performance liquid chromatography-tandem mass spectrometry (UPLC-MS/MS) method was developed and validated. Voriconazole was used as the internal standard to generate standard curves ranging from 5 to 1000 ng/mL. The separation was achieved using a UPLC system equipped with an Acquity UPLC BEH C_18_ column (2.1 × 50 mm, 1.7 μm) with 0.1% formic acid in acetonitrile and 0.1% formic acid in water as the mobile phase under gradient elution at a flow rate of 0.4 mL/min. The mass analysis was performed with a 4000 QTRAP^®^ mass spectrometer using multiple-ion reaction monitoring (MRM) in the positive mode, with the transition of *m*/*z* 325 → *m*/*z* 205 for OJT007 and *m*/*z* 350 → *m*/*z* 101 for voriconazole. The intra- and inter-day precision and accuracy were within ±15%. The mean extraction recovery and the matrix effect were 95.1% and 7.96%, respectively, suggesting no significant matrix interfering with the quantification of the drug in rat plasma. This study was successfully used for the pharmacokinetic evaluation of OJT007 using the rat as an animal model.

## 1. Introduction

Leishmaniasis is an infectious disease caused by protozoan parasites. The complex interaction between the parasite and the host immune cells causes heterogeneous clinical manifestations that range from localized skin lesions to widespread inflammatory responses [1]. Approximately 12 million people are infected with leishmaniasis, and the infection incidence is estimated at 2 million cases per year. Leishmaniasis is endemic across 98 countries on five continents, putting at risk at least 350 million people living in tropical areas [2]. Nevertheless, migration and travel have put previously unaffected populations at risk, such as the United States [3]. Cutaneous leishmaniasis (CL) is the most common form of leishmaniasis. In the Old World, Cutaneous Leishmaniasis is mainly caused by *Leishmania aethiopica*, *Leishmania tropica,* and *Leishmania major*. In the new world, it is caused by different species: *Leishmania braziliensis*, *Leishmania mexicana*, and *Leishmania venezuelensis* [4]. Currently, there are no available vaccines against leishmaniasis [5]. Furthermore, the available anti-leishmaniasis drugs suffer from toxic side effects [6], high cost [7], and drug resistance [8]. Miltefosine is the only approved oral drug for the treatment of leishmaniasis; nonetheless, it displays mixed efficacy and variability in clinical response [9,10]. Therefore, there is a critical need to develop oral drugs which are less toxic and more cost-effective for the treatment of leishmaniasis.

Methionine aminopeptidase (MetAP) is a metalloprotease which catalyzes the cleavage of N-terminal methionine from proteins during translation. There are two types of MetAPs, 1 and 2; prokaryotes have homologs of either type, while eukaryotes have both [11]. Nonetheless, eukaryotes develop lethal phenotypes if either MetAP gene is deleted [12]. N-terminal excision (NME) is necessary for post-translational modifications, stability, folding, and the localization of nascent proteins. The essentiality of NME in eukaryotes and prokaryotes makes it a promising target for infectious disease. Various types of MetAP have been validated as targets for a variety of infectious diseases caused by *Mycobacterium Tuberculosis* (*Mtb*, the causative agent of TB) [13], *Plasmodium falciparum* (the causative agent of malaria) [14], *Acinetobacter baumannii* (a Gram-negative bacillus resistant to most of the antibiotics in use) [15] and *Cryptosporidium parvum* (a parasite that infects epithelial cells and causes diarrhea) [16]. Recently, MetAP1 inhibitors have shown promising results against *Leishmania donovani*, giving more evidence for the use of MetAP1 inhibitors for the treatment of Leishmaniasis [17]. Hence, MetAP1 was selected as a chemotherapeutic target to find new antileishmanial treatments [18].

**Figure 1:**
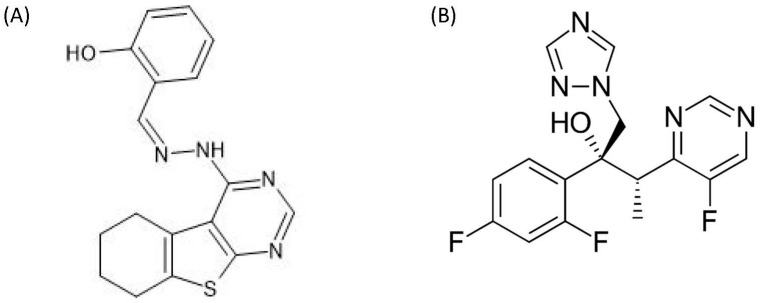
Chemical structure of (**A**) OJT007; (**B**) voriconazole (Internal Standard).

OJT007 (Figure 1; Panel A) is a MetAP1 inhibitor with potent and selective leishmanicidal activity against *Leishmania major*, the causative infectious agent of CL. This important lead molecule is a structurally diverse agent belonging to the hydrazine-1-ylidene-containing pharmacophore. OJT007 exhibited an EC_50_ of 500 nM against extracellular promastigotes and intracellular amastigotes, with little to no toxicity in mammalian cells. Likewise, the compound showed in vivo activity in a murine model [18]. These promising results make OJT007 an attractive candidate for the treatment of cutaneous leishmaniasis. However, in order to prepare this promising lead molecule for further clinical applications, preclinical studies of OJT007 are needed. The development and validation of bioanalytical assays are paramount during drug development to determine key parameters such as stability and pharmacokinetics.

In this study, we developed and validated a sensitive, specific, and reproducible ultra-high performance liquid chromatography-tandem mass spectrometry (UPLC-MS/MS) method for the quantification of OJT007 in rat plasma. The linearity, selectivity, sensitivity, and reproducibility were validated according to the FDA guidance for bioanalytical method validation. The stability of OJT007 in plasma was assessed according to the expected sample storage, handling, preparation, and analysis conditions. Subsequently, the validated method was successfully applied to pharmacokinetic studies of OJT007 in healthy male adult Sprague–Dawley (SD) rats, proving its suitability to characterize pharmacokinetic profiles following intravenous (IV) bolus drug administration.

## 2. Materials and Methods

### 2.1. Materials

The OJT007 (purity > 90%) was purchased from MolPort (Riga, Latvia). The voriconazole, formic acid, ascorbic acid and dimethyl sulfoxide (DMSO) were procured from Sigma-Aldrich (St. Louis, MO, USA). LC-MS grade water and acetonitrile were purchased from J.T. Baker Chemical Co. (Phillipsburg, NJ, USA). LC-MS/UPLC grade Methanol was acquired from Mallinckrodt Baker (Phillipsburg, NJ, USA). The blank rat plasma was purchased from Innovative Research (Novi, MI, USA). Male Sprague–Dawley (SD) rats were purchased from Envigo RMS (Indianapolis, IN, USA). All of the other chemicals and reagents were used as received.

### 2.2. Preparation of the Stock Solutions

The stock solution of OJT007 was prepared by dissolving the solid compound in 5% DMSO and 95% methanol at a concentration of 1 mg/mL, and was stored at −80 °C until it was used. Voriconazole was used as internal standard (IS) to analyze OJT007. The IS was prepared by dissolving voriconazole in acetonitrile at the concentration of 1 mg/mL. The OJT007 working solutions were prepared by diluting the stock solution in 50% acetonitrile in water at final concentrations of 10, 5, 2.5, 1, 0.5, 0.25, 0.10, and 0.05 μg/mL. The OJT007 quality control (QC) working solutions were prepared by diluting the stock solution in 50% acetonitrile in water at final concentrations of 0.15, 0.75 and 7.5 μg/mL. The IS working solution was prepared by diluting the 1 mg/mL stock solution with 100% acetonitrile to obtain a concentration of 300 ng/mL. A series of standard samples were prepared by spiking the working solutions (5 μL) into blank rat plasma (45 μL) containing 0.5% *w*/*v* ascorbic acid to obtain the following concentrations: 5, 10, 25, 50, 100, 250, 500, and 1000 ng/mL. The QC samples were prepared by spiking QC working solutions into blank rat plasma containing 0.5% *w*/*v* ascorbic acid to obtain concentrations of 15, 75 and 750 ng/mL.

### 2.3. Sample Preparation and Extraction

Plasma samples stored at −80 °C were thawed at room temperature. An aliquot (50 μL) of plasma was extracted for OJT007 using protein precipitation by adding 250 μL of acetonitrile containing the internal standard (300 ng/mL). The mixture was vortexed for 30 s and centrifuged at 14,000 rpm for 20 min at 4 °C. An aliquot of the supernatant was injected into the UPLC-MS/MS for quantitative analysis.

### 2.4. Instrument Conditions

The UPLC analysis was accomplished using a Shimadzu Nexera X2 UHPLC system (Columbia, MD, USA). The chromatographic separation was achieved using an Acquity UPLC BEH C_18_ column (50 × 2.1 mm, 1.7 μm) with a gradient mobile phase at flow rate of 0.4 mL/min. The sample injection volume was 10 μL, and the mobile phase consisted of 0.1% *v*/*v* formic acid in water (A) and 0.1% *v*/*v* formic acid in acetonitrile (B). Gradient elution was employed with 5% to 98% B from time 0 to 1.8 min, and was kept constant at 98% B for 1.7 min, then 98% B was changed to 5% B from 3.5 to 3.7 min, and kept constant at 5% from 3.7 min to 5 min.

The MS/MS analysis was performed on a 4000 QTRAP^®^ triple quadrupole mass spectrometer system equipped with a Turbo Ion Spray ion source (AB Sciex. Redwood City, CA, USA). The quantification was performed using multiple-ion reaction monitoring (MRM) in positive mode, with the transitions of *m*/*z* 324.9 → *m*/*z* 205.1 for OJT007 and *m*/*z* 350 → *m*/*z* 101 for IS. The source parameters were set as follows: ion spray voltage, 4500 V; ion source temperature, 500 °C; nebulizer gas, 50 psi; heater gas, 40 psi; curtain gas, 25 psi; and the collision gas, high. The compound-dependent parameters for OJT007 and IS were optimized with entrance potential (EP), 10 V and 10 V; declustering potential (DP), 105 V and 56 V; collision energy (CE), 30 V and 105 V; and collision cell exit potential (CXP), 11 V and 8 V, respectively. Analyst^®^ Software 1.6.2 (Redwood City, CA, USA) was used to control the UPLC-MS/MS system and analyze the data.

### 2.5. Validation

The UPLC-MS/MS assay was validated according to the Center for Drug Evaluation and Research (CDER) ‘Guidance for Industry: Bioanalytical Method Validation’ with regard to specificity, the lower limit of quantification (LLOQ), linearity and range, accuracy and precision, extraction recovery, matrix effect, carryover effect, stability and dilution integrity [19].

#### 2.5.1. Calibration Curve

Calibration curves in blank rat plasma were obtained by plotting the peak area ratio of OJT007 to IS versus the known concentration of OJT007. The least-squares linear regression method with 1/x^2^ weighting was applied in order to obtain the slope, intercept, and correlation coefficient. The LLOQ was evaluated based on a signal-to-noise ratio of at least 5:1. The LLOQ is the minimal quantifiable concentration point in the calibration curve at which the precision (coefficient of variation, CV) should be within 20%, and the accuracy (relative error, RE) should be within 20% of the nominal value.

#### 2.5.2. Stability in Plasma

The effect of ascorbic acid addition was evaluated by comparing the mean recovery percent of QC samples in the presence or absence of ascorbic acid. All of the stability studies were evaluated in blank rat plasma at low, medium, and high QC levels (15, 75, and 750 ng/mL, respectively) using triplicates at each concentration level. All of the samples were compared to freshly prepared samples at the same concentrations. Short-term QC samples in blank rat plasma were freshly prepared and left on the benchtop at room temperature for 4 h (short-term benchtop). The freeze–thaw (FT) stability samples were exposed to three cycles of freezing (−80 °C) and thawing (RT, room temperature). The stability of the processed samples was determined by comparing freshly obtained plasma extracts to plasma extracts that remained in the autosampler for 24 h at 15 °C. The long-term storage stability samples were freshly prepared and stored at −80 °C for 14 days.

#### 2.5.3. Extraction Recovery and Matrix Effect

In order to examine the extraction recovery and matrix effect, QC samples were evaluated at three concentration levels (15, 75 and 750 ng/mL, *n* = 6). The extraction recovery and matrix effect were calculated according to Equations (1) and (2).

(1) $$\mathrm{Recovery}\%\;=\frac{Response_{pre-extraction\;spike}}{Response_{post-extraction\;spike}}\times 100\%$$

where *Response_pre-extraction spike_* is the average area count for OJT007 that has undergone the extraction process, and *Response_post-extraction spike_* is for OJT007 samples spiked into the extracted matrix after the extraction procedure. The matrix factor of OJT007 was calculated according to:

(2) $$\mathrm{Matrix}\;\mathrm{Factor}\%\;=\left(\frac{Response_{post-extraction\;spike}}{Response_{matrix-free\;spike}}\times 100\%-1\right)$$

where *Response_post-extraction spike_* is the average area count for OJT007 spiked into the matrix after the extraction procedure, and *Response_matrix-free spike_* is the average area count for OJT007 samples at the same concentration of OJT007 in a neat solution (50% acetonitrile in water).

#### 2.5.4. Accuracy and Precision

In order to evaluate the intra-day and inter-day assay accuracy and precision, the LLOQ and QC samples were analyzed on the same day or three subsequent days. The experiments were conducted in sextuplicates. The assay accuracy and precision were expressed in terms of the relative error from the theoretical drug concentration (RE%) and coefficient of variation (CV%), respectively.

#### 2.5.5. Dilution Integrity

The dilution integrity was assessed by determining the accuracy and precision of the measurement of rat plasma spiked with an OJT007 5X upper limit of quantification (ULOQ). The effect of 1:5, 1:10, 1:20 and 1:50 dilution with blank rat plasma was measured. Following the UPLC-MS/MS analysis, the quantitated concentrations were corrected with their respective dilution factor, and the accuracy and precision were determined. This experiment was conducted in sextuplicate.

### 2.6. Pharmacokinetic Study

The validated assay was applied to a pharmacokinetic study for the quantification of OJT007 in a biological matrix. The study in rats was performed using the protocol approved by the Texas Southern University Animal Care and Use Committee (#9068). All of the experimental procedures were performed in accordance with the ‘National Institute of Health: Guide for the Care and Use of Laboratory Animals’ [20]. A single-dose study design was used to evaluate the pharmacokinetics of OJT007 following intravenous administration. Three jugular vein-cannulated, healthy, adult, male Sprague–Dawley rats received a 5 mg/kg intravenous dose of OJT007 cosolvent formulation. Serial blood samples (0.2 mL) were collected before dosing, and at 0.033, 0.0833, 0.25, 0.5,1, 1.5, 2, 4, 6, 8, 10, and 24 h post-dose. The blood samples were placed into heparinized Eppendorf tubes and centrifuged at 14,000 rpm for 3 min. The plasma samples were collected into an Eppendorf tube containing 0.5% *w*/*v* ascorbic acid and stored at −80 °C until their analysis. The plasma samples were analyzed within 14 days. The plasma concentration versus time profiles were analyzed for each rat using noncompartmental analysis (WinNonlin v8.1, Pharsight Corp, Mountain View, CA, USA). The pharmacokinetic parameters derived were terminal elimination half-life (t_1/2_), plasma clearance (CL), apparent volume of distribution at steady-state (V_ss_), Mean Residence Time (MRT), the area under the curve to the last quantified time (AUC_0–last_), and the area under the curve extrapolated to infinity (AUC_0–∞_), as calculated using a log-linear trapezoid method.

### 2.7. Statistical Analysis

The plasma concentration data are presented as the mean value with standard deviation (SD). SigmaPlot (SYSTAT Software, San Jose, CA, USA) was used for the curve fitting.

## 3. Results and Discussion

### 3.1. UPLC-MS/MS Method Development

#### 3.1.1. Mass Conditions

**Figure 2:**
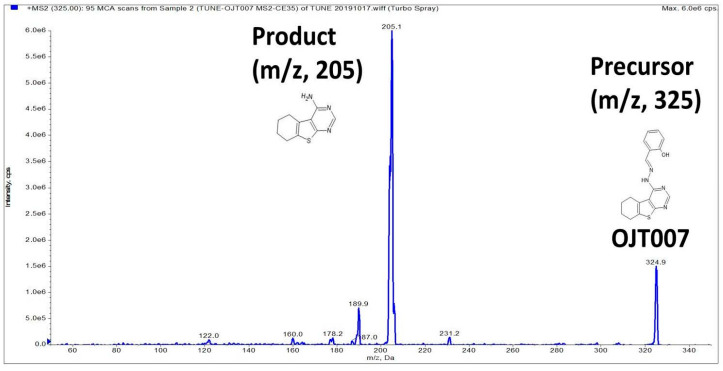
Product ion scan spectra for OJT007 (*m*/*z* 324.9→205.1).

The positive ion mode and electrospray ionization (ESI) were found to provide the best peak intensity compared to the negative mode and atmospheric chemical ionization (APCI). After the collision cell fragmentation, the most abundant and stable MRM transition was 205 for OJT007. The MS fragmentation pattern of OJT007 is shown in Figure 2. The source parameters were optimized in order to obtain the most suitable conditions for the analyte, ensuring signal stability and sensitivity.

#### 3.1.2. Chromatographic Conditions

**Figure 3:**
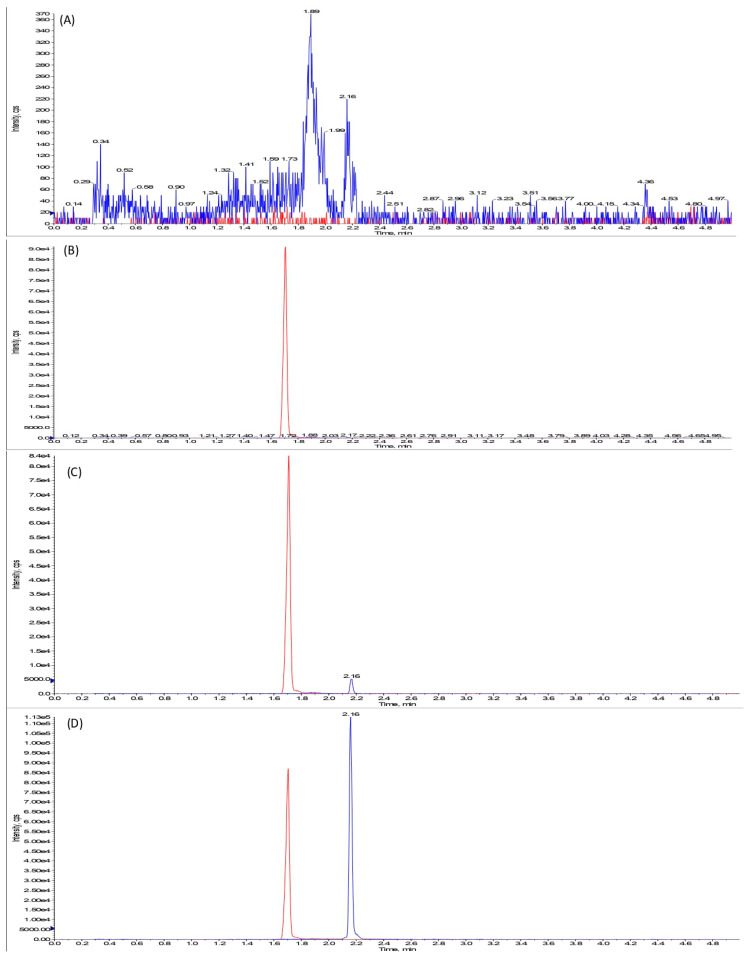
Representative chromatograms of OJT007 and voriconazole (IS) in rat plasma: (**A**) blank rat plasma; (**B**) blank rat plasma spiked with IS; (**C**) blank rat plasma spiked with IS and OJT007 at 5 ng/mL; (**D**) blank rat plasma spiked with IS and OJT007 at 100 ng/mL.

OJT007 does not have a commercially available stable isotope-labeled analog. As such, we selected voriconazole as the internal standard, which has a similar ionization mode, sensitivity, and lipophilicity to OJT007 [21]. Several mobile phase compositions and columns were tested until an optimal intensity and peak shape were attained. The best response and peak shape were achieved with an Acquity UPLC BEH C_18_ column (50 × 2.1 mm, 1.7 μm) using 0.1% formic acid in acetonitrile and water as the mobile phase. OJT007 is a weak base analyte; the addition of 0.1% formic acid to the mobile phase aids in the formation of ions in the solution in order to achieve the optimal response in ESI. Although the analyte was in an ionized form, an adequate chromatographic performance was observed. This may be due to the analyte’s hydrophobic moiety maintaining the ability to interact with the stationary phase. A good peak shape may be attributed to the Acquity UPLC BEH C_18_ column’s endcapping, effectively eliminating unbounded silanol groups preventing ionic interactions with the positively charged analyte. The gradient was optimized to ensure appropriate chromatographic retention and separation while preventing carryover. Figure 3 illustrates the chromatograms obtained under the established chromatographic conditions. The blank rat plasma chromatogram (Figure 3; Panel A) demonstrates the absence of interference at the time of elution for OJT007 and IS. The chromatographic run time for the developed assay was 5 min, with retention times of 2.16 and 1.74 for OJT007 and IS, respectively (Figure 3; Panel C and D). Potential carryover interference peaks between the injections were prevented using an organic solvent mixture comprising 50% acetonitrile and 50% methanol as a needle wash between the injections.

### 3.2. Method Validation

#### 3.2.1. Linearity and Sensitivity

The standard curves of OJT007 in plasma were linear in the concentration range of 5–1000 ng/mL. Linear correlation coefficients greater than 0.99 were considered acceptable for the quantification of the analyte in the biological matrix. Based on a signal-to-noise ratio of at least 5:1, the LLOQ for the developed assay was 5 ng/mL in rat plasma.

#### 3.2.2. Stability

Ascorbic acid (0.5% *w*/*v*) was added to the OJT007 plasma samples to prevent oxidation and ensure the stability of the OJT007. The effect of ascorbic acid in rat plasma is summarized in Table 1. The addition of ascorbic acid to the plasma samples increased their short-term stability and freeze–thaw stability by 20%. These results suggest that the addition of ascorbic acid could prevent drug degradation during analysis. Small molecules containing phenol groups are readily oxidized in biological matrices [22]. Ascorbic acid is a reducing agent which is readily oxidized [23]. OJT007 contains a phenol group in its chemical structure. Ascorbic acid may act as a sacrificial agent, preventing the oxidation of OJT007. Nonetheless, further studies are required in order to understand the oxidation process of OJT007.

Stability studies were conducted to evaluate the stability of the analyte under the expected storage and handling conditions. The stability study results—expressed as the mean remaining percentage of the nominal concentration—are summarized in Table 2. OJT007 was stable for up to 4 h in rat plasma at room temperature, eliminating any concern regarding analyte degradation during sample preparation. The long-term stability samples at low, medium, and high QC concentrations were within 10% of the nominal concentration, demonstrating that pharmacokinetic samples could be stored at −80 °C for up to 14 days without degrading the integrity of the sample. The mean recovery of OJT007 from rat plasma following three freeze–thaw cycles was 95.1%, 98.3% and 96.1% at low, medium, and high QC samples, respectively, suggesting that repeatedly freeze–thawing the plasma samples did not influence the OJT007’s stability. The autosampler stability was expressed as the mean recovery OJT007 from the extracted plasma placed on the autosampler for up to 24 h at 15 °C (Table 2). The data demonstrate that OJT007 is stable in processed samples during analysis conditions for up to 24 h.

#### 3.2.3. Extraction Recovery and Matrix Effect

The extraction recovery of OJT007, expressed as the percentage recovered (Table 3), was calculated as a ratio of the response obtained from a sample of the biological matrix with OJT007 before protein precipitation to the response from sample spiked after protein precipitation. The mean extraction recovery for OJT007 was 95.8% at the low QC (15 ng/mL), 98.3% at the medium QC (75 ng/mL), and 91.2% at the high QC concentration (750 ng/mL). This data indicates that the OJT007 extraction method from plasma by protein precipitation with acetonitrile is appropriate.

The matrix effect indicates potential ion enhancement or suppression caused by co-eluting matrix components during UPLC-MS/MS analysis. A positive matrix factor value signifies an enhancement of the analyte signal, while a negative value indicates analyte signal suppression. No significant matrix effect is considered to have taken place if the matrix factor is within ±15%. Table 3 shows the average matrix factors for low, medium, and high QC concentrations of OJT007 in plasma. This data suggests that the sample preparation method successfully removes the matrix components responsible for variability in the analyte signal intensity. Furthermore, this data suggests that the chromatographic conditions effectively prevented ion suppression or enhancement.

#### 3.2.4. Accuracy and Precision

The intra- and inter-day accuracy, presented as the percentage of relative error, RE%; and precision, presented as the percentage coefficient of variation, CV%, are summarized in Table 4. The intraday coefficient of variation ranged from 1.96 to 8.28%, and the percentage of relative error ranged from 3.40 to 10.1% in plasma. The inter-day coefficient of variation ranged from 3.99 to 11.5%, and the percentage of relative error ranged from 5.45 to 9.69% in plasma. The precision and accuracy were within the acceptable limit of ≤20% for LLOQ and ≤15% for the high, medium, and low QC, as established by the FDA Bioanalysis guidance. This data indicates that the developed UPLC-MS/MS method is accurate and precise for the quantification of OJT007 in rat plasma over a concentration range of 5–1000 ng/mL.

#### 3.2.5. Dilution Integrity

The dilution integrity was assessed by determining the accuracy and precision of the samples diluted with blank plasma. The accuracy (RE%) was less than 7.35%, and the precision (CV%) was less than 8.91% (Table 5). The data suggest that the concentration of OJT007 in plasma samples of greater concentrations than the ULOQ can be accurately and precisely quantified following up to 50 times dilution with blank plasma before the analysis.

### 3.3. Pharmacokinetic Studies

**Figure 4:**
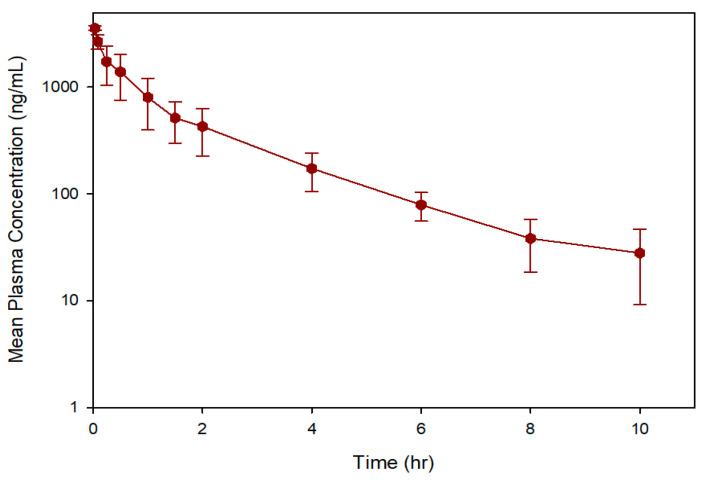
Mean ± SD plasma concentration-time profile of OJT007 after a single 5 mg/Kg intravenous dose to male Sprague-Dawley rats.

The validated UPLC-MS/MS method was successfully applied to the pharmacokinetic study of OJT007 following the intravenous administration of a single dose of 5 mg/kg. The plasma concentration versus time profiles for OJT007 following the OJT007 administration are shown in Figure 4. The pharmacokinetic parameters for OJT007 in rats are summarized in Table 6. OJT007 displayed a biexponential disposition after intravenous administration, with a rapid distribution followed by a slow elimination. The mean volume of distribution at a steady state (V_ss_) was 4.93 L/kg, which is greater than the total body water in rats [24], possibly due to drug partition into tissues. The mean systemic clearance (CL) was 2.31 L/h/kg, which approximates rats’ hepatic blood flow [24], suggesting high liver extraction. The mean plasma concentration–time curve during the period of observation (AUC_0→last_) and the area under the plasma concentration–time curve extrapolated to infinity (AUC_0→∞_) were 3.12 mg.h/L and 3.18 mg.h/L, respectively. The terminal elimination half-life and mean residence time were 1.86 h and 1.98 h, respectively. One limitation of the study is its relatively small sample size. Further studies are warranted to study OJT007 metabolism mechanisms and oral bioavailability.

## 4. Conclusions

A sensitive, specific, and reproducible UPLC-MS/MS method was developed and validated to quantify OJT007 in rat plasma. This method was successfully used for pharmacokinetic studies. This method was shown to be linear, accurate, and precise over the concentration range 5–1000 ng/mL. The method displayed good recovery without interference from endogenous components in the plasma. OJT007 remains stable under the expected sample handling, storage, preparation, and analysis conditions. This assay was successfully applied to a single dose pharmacokinetic study using SD rats as an animal model, revealing that OJT007 has a biexponential disposition in rats after intravenous administration. This method can be applied to future studies of OJT007.

## Figures and Tables

**Table 1:** Mean recovery of OJT007 in the presence or absence of ascorbic acid in rat plasma.

Stability Test	Ascorbic Acid	Mean Recovery%
Short-Term Stability (4 h, RT)	No	76.7 ± 11.2
	Yes	97.0 ± 5.9
Long-Term Stability (14 days, −80 °C)	No	93.7 ± 8.8
	Yes	94.8 ± 3.2
Auto-Sampler Stability (24 h)	No	98.0 ± 11.7
	Yes	96.1 ± 7.9
Freeze-Thaw (−80 °C to RT)	No	70.7 ± 4.4
	Yes	96.5 ± 1.6

**Table 2:** Stability of OJT007 in rat plasma, expressed as a percentage of the nominal concentration [*n* = 3; mean (±SD)].

Stability Test	Nominal Concentration (ng/mL)	Mean Recovery% ± SD
Short-Term Stability (4 h, RT)	15	102.4 ± 4.4
	75	97.1 ± 3.3
	750	91.4 ± 3.9
Long-Term Stability (14 days, −80 °C)	15	96.0 ± 4.3
	75	93.8 ± 1.4
	750	94.7 ± 3.9
Auto-Sampler Stability (24 h)	15	89.6 ± 7.7
	75	97.2 ± 8.7
	750	101.6 ± 1.9
Freeze Thaw Stability (−80 °C to RT)	15	95.1 ± 0.8
	75	98.3 ± 0.4
	750	96.1 ± 1.2

**Table 3:** Extraction recovery rates and matrix factors of the UPLC-MS/MS method for the analysis of OJT007 in rat plasma [*n* = 6; mean (± SD)].

Nominal Concentration (ng/mL)	Extraction Recovery (Mean ± SD, %)	Matrix Effect (Mean ± SD, %)
15	95.8 ± 1.5	3.79 ± 0.53
75	98.3 ± 1.7	8.73 ± 1.71
750	91.2 ± 2.4	3.40 ± 1.36

**Table 4:** Intra- and inter-day accuracy and precision of the UPLC-MS/MS method for the quantification of OJT007.

QC	Nominal Concentration (ng/mL)	Intra-Day (*n* = 6)		Inter-Day (*n* = 6)	
		Accuracy (RE, %)	Precision (CV, %)	Accuracy (RE, %)	Precision (CV, %)
LLOQ	5	5.60	8.28	9.69	11.5
Low	15	10.1	3.49	5.78	7.29
Medium	75	2.88	1.96	5.52	4.58
High	750	3.40	3.03	5.45	3.99

**Table 5:** Dilution integrity accuracy and precision of OJT007 in rat plasma.

Nominal Concentration (ng/mL)	Dilution Factor	Accuracy (RE%) (*n* = 6)	Precision (CV%) (*n* = 6)
5000	5	7.35	5.67
	10	6.87	3.66
	20	3.77	8.91
	50	7.03	4.95

**Table 6:** Pharmacokinetic parameters (Mean ± SD) after the intravenous administration of 5 mg/Kg OJT007 to male Sprague-Dawley rats (*n* = 3).

Parameters	Mean ± SD
AUC_0–10_ (mg.h/L) *	3.12 ± 1.32
AUC_0–∞_ (mg.h/L) *	3.18 ± 1.36
T_1/2_ (h) *	1.86 ± 0.22
CL (L/h/kg) *	2.31 ± 0.90
Vss (L/kg) *	4.93 ± 2.00
MRT (h) *	1.98 ± 0.03

* AUC_0–last_(mg.h/L) = area under the curve from 0 to the last measured concentration; AUC_0–>∞_ = area under the curve from 0 to ∞; T_1/2_ = terminal elimination half-life; CL = total clearance; V_ss_ = volume of distribution at a steady state; MRT = mean residence time.

## Data Availability

The data presented in this study are available within this article.
